# The safety of combining Endostar with concurrent chemoradiotherapy for the treatment of locally advanced cervical cancer and the evaluation of its anti-angiogenic effects via transrectal contrast-enhanced ultrasound

**DOI:** 10.3389/fonc.2025.1514425

**Published:** 2025-05-08

**Authors:** Fang Wu, Zhouxue Lu, Jinting Que, Shanshan Ma, Li Jiang, Xiaobi Tang, Chengshan Zheng, Li Zhou, Qiufeng Huang, Yong Zhang

**Affiliations:** ^1^ Department of Radiation Oncology, The First Affiliated Hospital of Guangxi Medical University, Nanning, Guangxi, China; ^2^ Key Laboratory of Early Prevention and Treatment for Regional High Frequency Tumor, Guangxi Medical University, Ministry of Education, Nanning, Guangxi, China; ^3^ Department of Oncology, The First People’s Hospital of Qinzhou, Qinzhou, Guangxi, China

**Keywords:** safety, antiangiogenic, Endostar, cervical cancer, transrectal contrast-enhanced ultrasound

## Abstract

**Background:**

In recent years, exploring the addition of angiogenesis inhibitors to chemoradiotherapy for locally advanced cervical cancer (LACC) has gained research interest. This study assessed the safety and anti-angiogenic effects of combining Endostar with concurrent chemoradiotherapy (CCRT) via transrectal contrast-enhanced ultrasound.

**Methods:**

A total of 120 patients with locally advanced cervical cancer (LACC) were randomly allocated to two groups: CCRT combined with Endostar (CRT+E group, n = 60) and CCRT alone (CRT group, n = 60). Endostar was administered intravenously before radiotherapy and repeated for four cycles. All patients received platinum-based CCRT. Adverse events were monitored, and transrectal contrast-enhanced ultrasonography (CEUS) was conducted before, during, and after radiotherapy. Vascular malformation (VM) rates were calculated from tumor cross-sectional images, and quantitative analysis software measured peak intensity (PI), time to peak (TTP), and mean transit time (MTT) of tumor vessels.

**Results:**

No significant differences were observed in hematological, hepatic, renal, gastrointestinal, or cardiac adverse reactions between the two groups (all P>0.05). In the CRT+E group, VM rates, TTP, and MTT significantly differed at three time points (with P values of 0.003, 0.002, and P<0.001, respectively), whereas the CRT group showed no significant changes (all P>0.05). Post-radiotherapy, statistically significant differences emerged between the CRT+E and CRT groups for VM rates (P = 0.027), MTT (P = 0.027), and TTP (P < 0.001), while PI showed no significant difference (65.67 ± 36.53 vs. 74.69 ± 61.21, P = 0.598).

**Conclusion:**

The combination of Endostar with CCRT for locally advanced cervical cancer (LACC) demonstrated favorable safety and tolerability. Transrectal contrast-enhanced ultrasound (CEUS) effectively assessed tumor vascular normalization induced by Endostar during CCRT. Specifically, Endostar significantly reduced VM rates and shortened MTT, suggesting its potential to normalize tumor vasculature.

## 1 Introduction

Cervical cancer (CC) is the most common malignancy of the reproductive system in women, ranking as the fourth most frequently diagnosed cancer and the fourth leading cause of cancer death in women worldwide (1, 2). Statistics show that there were approximately 604,000 new cases and 342,000 deaths globally in 2020, with more than 70% of new cases and 90% of deaths occurring in developing countries (1, 3). More than 70% of CC patients present with locoregionally advanced disease (LACC), which is associated with poor prognosis. Platinum-based concurrent chemoradiotherapy (CCRT) is considered the standard treatment for LACC since 1999. However, 30% - 50% of patients experience disease recurrence, including distant metastatic progression despite achieving local control post-CCRT (4).

Endostar, a recombinant human endostatin independently developed and patented in China, inhibits vascular endothelial cell migration to block the formation of tumor neovascularization, which plays an anti-angiogenic role in multiple targets and has no drug resistance. Previous researches demonstrated that Endostar restricts angiogenesis by blocking VEGF-induced tyrosine phosphorylation of the membrane surface receptor KDR/flk-1(VEGFR-2, vascular endothelial growth factor receptor-2) and inducing apoptosis via caspase-3 activation coupled with Bcl-2 downregulation in human umbilical vein endothelial cells (HUVECs) (5, 6). As an anti-angiogenic agent, Endostar can improve the abnormal tumor structure, increase vascular perfusion within the validity of the agent. This process, termed tumor vascular normalization, improves the efficacy of radiotherapy and chemotherapy (7, 8). Both clinical studies and standard practice have established the efficacy of antiangiogenic strategies in cancer treatment, particularly through angiogenesis inhibitors. Recent research has increasingly focused on integrating these inhibitors with chemoradiotherapy for LACC. This combined approach (antiangiogenic therapy + CCRT) demonstrates promising potential to improve LACC patient outcomes (9). In 2005, the Chinese Food and Drug Administration (CFDA) formally approved Endostar as a first-line therapy for recurrent and metastatic non-small cell lung cancer (10). Currently, studies involving non-small cell lung cancer (10), nasopharyngeal carcinoma (11), bone and soft tissue sarcomas (12) have shown synergistic effects of Endostar when combined with radiotherapy and chemotherapy. However, evidence supporting its role in CC remains limited.

While the theory of vascular normalization is widely recognized, there is a lack of comprehensive clinical evaluation in CC. Dynamic contrast-enhanced ultrasound (DCE-US), a recently developed imaging modality, enables quantitative assessment of solid tumor perfusion through analysis of raw linear data, providing a promising avenue for evaluating anti-angiogenic therapy in various malignant tumors (13). DCE-US offers distinct advantages: non-invasiveness, portability, cost-effectiveness, high sensitivity, and reproducibility, coupled with minimal radiation exposure and low risk of allergic reactions compared to iodinated contrast agents (14, 15). Previous studies have demonstrated that transrectal ultrasound produces high-resolution images of the cervix, uterus, and adjacent structures, and when combined with contrast agents, it enables detailed evaluation of tumor perfusion dynamics (16–18). Although transvaginal contrast-enhanced ultrasound (CEUS) is widely used for assessing gynecologic tumors, many patients with LACC develop cervical stenosis or anatomical distortion due to tumor infiltration, making transvaginal access challenging or even unfeasible. In contrast, transrectal CEUS is a well-established technique for evaluating gynecologic and pelvic malignancies. It has been shown to provide reliable vascular and perfusion-related parameters, making it a valuable tool for assessing treatment response in LACC (19). Therefore, this study provides novel insights into the safety of Endostar in combination with CCRT compared to CCRT alone for treating LACC. Furthermore, we quantitatively evaluated the anti-angiogenic efficacy of this combination using transrectal contrast-enhanced ultrasound (CEUS) to provide clinical evidence to optimize therapeutic protocols for LACC.

## 2 Materials and methods

The present study was a parallel, randomized, controlled clinical trial for LACC clinical treatment. The protocol of the present study was approved by the Ethics Committee at the First Affiliated Hospital of Guangxi Medical University (2023-E662-01). Written informed consent was obtained from all patients. The study protocol strictly followed the Declaration of Helsinki.

### 2.1 Sample size estimation

In this study, an independent t-test was used to compare the differences in CEUS parameters between the two groups. Preliminary experiments revealed that the mean MTT value after radiotherapy was 71.12 ± 33.95 in the Endostar plus CRT (CRT+E) group, compared to 55.47 ± 19.20 in the CRT group. Using G*Power software (version 3.1.9.7; Heinrich Heine University, Düsseldorf, Germany), we calculated that 92 patients (46 per group) would provide 85% power to detect this difference at a two-sided significance level of α = 0.05. To account for an anticipated 15% dropout rate and loss to follow-up, the final sample size was inflated to 120 patients (60 per group).

### 2.2 Patients

Consecutive patients with LACC at the first affiliated hospital of Guangxi Medical University were enrolled from March 2017 to September 2020. The inclusion criteria were as follows: age 18 to 75 years; KPS (Karnofsky Performance Status) score ≥ 70 points; pathologically confirmed patients with FIGO stage IB2, IIA2-IVA (2009 FIGO staging) CC; with evaluable tumor lesions; no previous radiotherapy or chemotherapy; without serious liver, kidney, and other organ dysfunction; at least 6 months of expected survival time. The exclusion criteria were as follows: patients with distant metastases; patients with other malignant tumors; patients unable to tolerate chemoradiotherapy or targeted therapy, including serious cardiovascular disease, serious liver or kidney failure, serious neurological or mental deficiency, and acute infectious diseases; patients who received anti-tumor therapy previously; pregnant or lactating patients; those who have received targeted therapy; those undergoing trials for other drugs.

### 2.3 Treatment

Eligible patients were randomly allocated (1:1) to receive CCRT plus Endostar (CRT+E group) or CCRT alone (CRT group).

### 2.4 CCRT for both groups

All patients received CCRT, comprising radiotherapy with weekly cisplatin (40 mg/m²). External beam radiotherapy (EBRT) was delivered via intensity-modulated conformal radiotherapy (IMRT) using a 6-MV X-ray linear accelerator, prescribed to 45–50 Gy in 25 fractions (1.8-2.0 Gy/fraction), administered 5 times weekly. Metastatic pelvic lymph nodes received simultaneous integrated boost (SIB) to 55–60 Gy in 25 fractions (2.2-2.4 Gy/fraction). Target volumes and organs at risk were delineated using pelvic MRI, supplemented by gynecological examination findings. Following EBRT, high-dose-rate intracavitary brachytherapy (HDR-ICBT) was performed twice weekly, delivering 6–7 Gy per fraction to point A, with a total dose of 28–30 Gy in 4–5 fractions. For patients unsuitable for brachytherapy (e.g., cervical stenosis or extensive parametrial involvement), a SIB to the primary tumor was utilized as an alternative (20). Concurrent chemotherapy consisted of intravenous cisplatin (40 mg/m²) administered weekly for 4–5 cycles, starting on day 1 of radiotherapy. Routine prophylactic use of antiemetic drugs was provided during chemotherapy.

### 2.5 Endostar therapy for the CRT+E group

Endostar (Simcere Pharmaceutical Group, Nanjing, China) was administered at 7.5 mg/m²/d via continuous intravenous infusion for 10 days, starting 5 days before the initiation of radiotherapy. The regimen was repeated every 15 days for 4 cycles.

### 2.6 Transrectal contrast-enhanced ultrasound examination and parametric CEUS analysis

All patients underwent transrectal CEUS before, during and after radiotherapy to assess tumor blood perfusion. CEUS parameters were used to monitor changes in tumor vasculature and blood flow over time.

CEUS examination method: Ultrasound imaging was performed using an Aplio 500 color Doppler ultrasound diagnostic system (Toshiba, Tokyo, Japan) equipped with a 2.5–8.5 MHz probe. This system features tissue imaging (DTHI), contrast-enhanced ultrasound, and advanced dynamic flow imaging capabilities. Additionally, it supports dynamic imaging data storage (SDID), enabling repeated data analysis. The contrast agent SonoVue was prepared by adding 5 mL of 0.9% normal saline to a 59 mg SonoVue bottle to generate sulfur hexafluoride microbubbles. The solution was mixed thoroughly, and a fixed volume of 2.4 mL of the microbubble suspension was extracted using a syringe. The contrast agent was injected into the patient’s left antecubital vein via a pre-installed indwelling needle at a controlled rate of 1 mL/sec. Immediately after injection, a 5 mL saline flush was administered to ensure complete delivery. Simultaneously, a timer was started at the moment of contrast injection, and a CEUS dynamic video of at least 60 seconds was recorded in Audio Video Interleaved (AVI) format for future analysis. By capturing vascular reconstruction images of the tumor’s maximum cross-section, associate chief physicians or senior attending physicians, with at least 5 years of DCEUS experience and an annual caseload of more than 500 patients, assess the tumor’s blood vessels using the same ultrasound diagnostic instrument. To ensure the objectivity and validity of imaging evaluation, all CEUS image analyses were performed blindly, with sonographers blinded to patients’ treatment group assignments. Pathological vessels with irregular, cystic, or sinusoidal shapes were categorized as vascular malformations (VM), and the total number of vessels on the maximum cross-section was recorded to calculate the VM rate (malformation vessel number/total vessel number). Quantitative analysis was performed using contrast-enhanced ultrasound quantitative analysis software, and three parameters including peak intensity (PI), time to peak (TTP), and mean transit time (MTT) were obtained. These parameters were recorded before, during, and after radiotherapy.

### 2.7 Adverse reaction assessment

Treatment-related adverse events (AEs), including chemotherapy-associated and radiation-induced complications, were evaluated weekly during treatment. Chemotherapy-related AEs were graded according to the National Cancer Institute’s Common Terminology Criteria for Adverse Events (CTCAE) v5.0 (Grade 1-5). Radiation-induced toxicities were evaluated using the Radiation Therapy Oncology Group (RTOG) morbidity scoring criteria, with severity categorized as Grade 0 (none) to Grade 4 (life-threatening).

### 2.8 Statistical analysis

The data were analyzed using SPSS 25.0 (IBM Corporation, Armonk, NY, USA). The measurement data were expressed as mean ± SD. The t-test was used to compare means between different groups, while repeated measures analysis of variance and the Bonferroni t-test were employed to analyze continuous variables within the same group. The rates of the two groups were compared using Chi-square analysis or Fisher’s exact probability method. Statistical significance was set at P<0.05.

## 3 Results

### 3.1 Patients

A total of 120 patients with LACC were enrolled and randomly assigned (1:1) to either the CRT+E group (n=60) or the CRT group (n=60). The median age was 53 years (range: 30–65 years), with 90.8% (109/120) diagnosed with squamous cell carcinoma and 9.2% (11/120) with adenocarcinoma. FIGO 2009 staging distribution was: IB2 (n=5, 4.2%), IIA2 (n=16, 13.3%), IIB (n=78, 65.0%), IIIA (n=4, 3.3%), IIIB (n=13, 10.8%), and IVA (n=4, 3.3%). There was no statistically significant difference between the two groups in terms of baseline characteristics, including age, staging, and histology. See **Table 1** for details.

**Table 1:** Baseline characteristics of patients.

Characteristics	CRT+E n=60	CRT n=60	*P-value*
Age (years)	53.60 ± 8.11	52.47 ± 7.52	0.97
FIGO staging^a^			0.854
IB2	3	2	
IIA2	9	7	
IIB	40	38	
IIIA	1	3	
IIIB	5	8	
IVA	2	2	
histopathologic type			0.752
Squamous	55	54	
adenocarcinoma	5	6	

a Staging according to the 2009 of FIGO staging for cervical cancer; P-values were calculated using either the Fisher’s exact test or chi-squared test.

CRT+E, simultaneous radiotherapy and chemotherapy combined with Endostar; CRT, simultaneous radiotherapy and chemotherapy.

### 3.2 Therapy related AEs

Following the CTCAE 5.0 and the RTOG radiation reaction assessment criteria, we evaluated adverse reactions during treatment. It is noteworthy that all enrolled patients successfully adhered to the prescribed treatment plan and managed the resulting adverse reactions with the help of appropriate support and symptomatic care. In both the CRT+E and the CRT groups, the most frequently observed adverse reactions included hematological toxic reactions and gastrointestinal toxic reactions, with incidence rates of 91.6% vs. 90% (P=0.945) and 76.6% vs. 75% (P=0.937), respectively. Notably, Grade 1–2 toxic reactions were observed in 51.6% vs. 43.3% (P=0.585) and 70.0% vs. 66.7% (P=0.865), while Grade 3–4 toxic reactions were found in 40.0% vs. 46.7% (P=0.643) and 6.7% vs. 8.3% (P=0.784). Importantly, the observed differences were not statistically significant. The statistical data are described in **Table 2**.

**Table 2:** The occurrence and comparison of acute toxic and side effects between the two groups.

Adverse reaction	CRT+E (n=60)			CRT (n=60)			*X2*	*P-value*
	All	Grade1-2	Grade3-4	All	Grade1-2	Grade3-4		
Hematological toxicity	55	31	24	54	26	28	0.005	0.945
Hepatic toxicity	5	5	0	5	5	0	0	1
Renal toxicity	5	5	0	2	2	0	0.607	0.436
Cardiac toxicity	0	0	0	0	0	0	0	1
Gastrointestinal toxicity	46	42	4	45	40	5	0.006	0.937

P-values were calculated by Chi-square analysis.

### 3.3 Transrectal contrast-enhanced ultrasound parametric analysis

**Figure 1:**
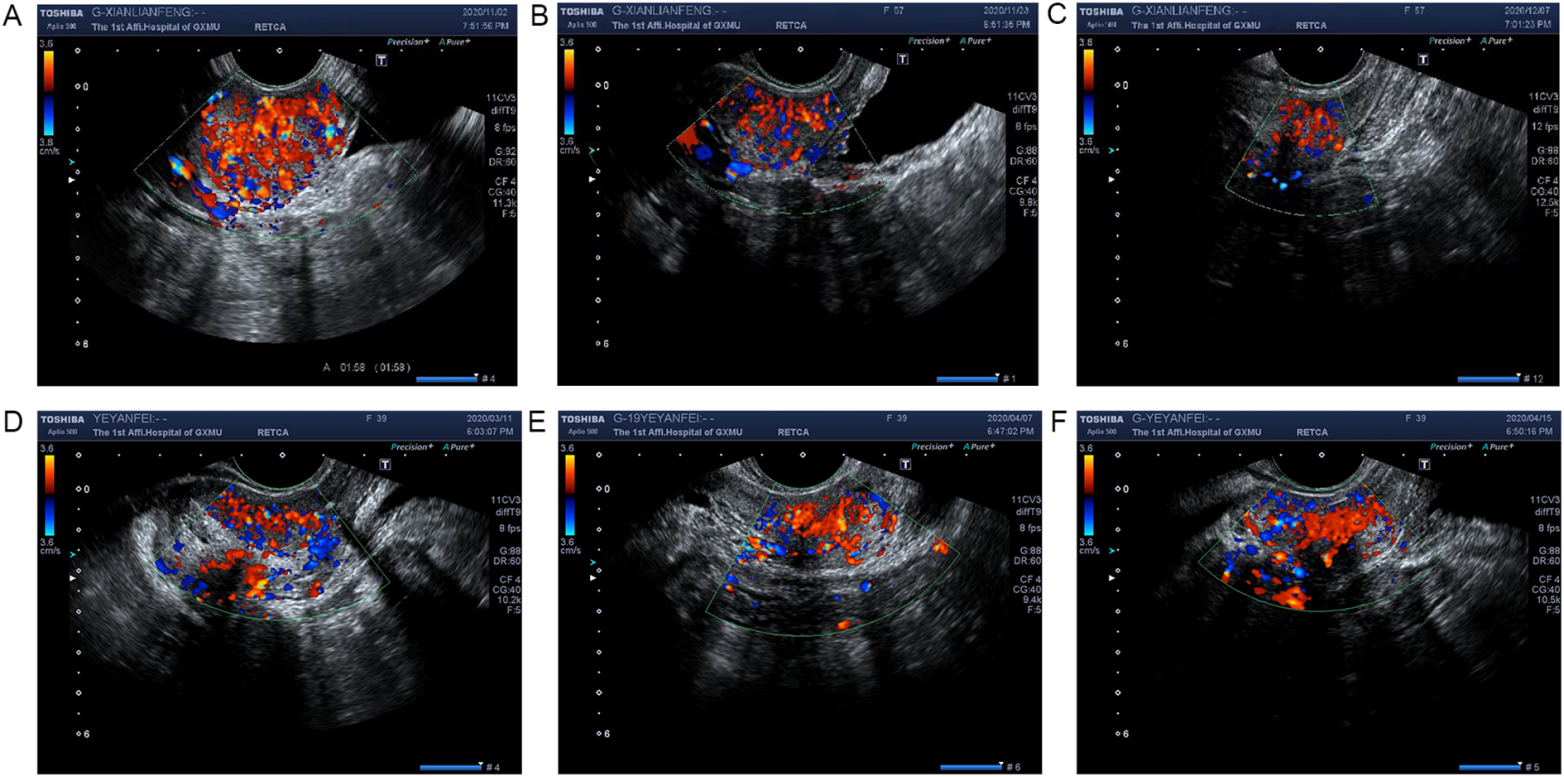
Color Doppler ultrasonography imaging of cervical cancer. **(A–C)** Representative color Doppler ultrasonography images of cervical cancer before **(A)**, during **(B)**, and after **(C)** radiotherapy in patients of the CRT+E group, demonstrating changes in tumor vascularity over the course of treatment. **(D–F)** Representative color Doppler ultrasonography images of cervical cancer before **(D)**, during **(E)**, and after **(F)** radiotherapy in patients of the CRT group, showing differences in vascular morphology and perfusion compared to the CRT+E group.

**Figure 2:**
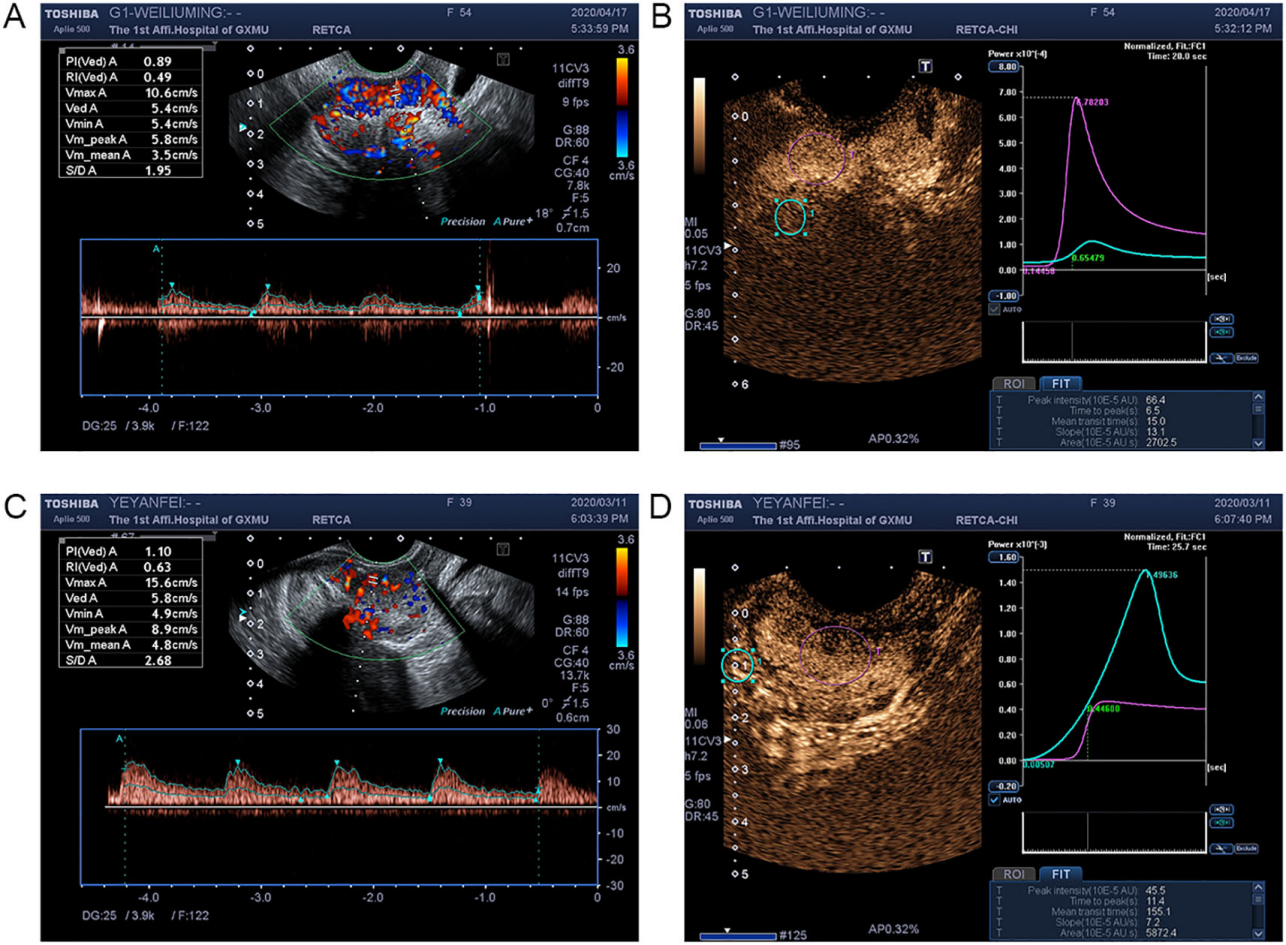
DCE-US hemodynamic resistance index imaging and time-intensity curves in cervical cancer patients. **(A)** Representative DCE-US hemodynamic resistance index imaging in patients from the CRT+E group, illustrating vascular perfusion characteristics. **(B)** Time-intensity curves for the CRT+E group, with green representing normal tissue and red representing the tumor, demonstrating changes in contrast enhancement over time. **(C)** Representative DCE-US hemodynamic resistance index imaging in patients from the CRT group, showing vascular perfusion differences compared to the CRT+E group. **(D)** Time-intensity curves for the CRT group, with green representing normal tissue and red representing the tumor, highlighting variations in hemodynamic response.

To evaluate the anti-angiogenic efficacy, transrectal color Doppler ultrasonography was performed at three time points: before, during, and after radiotherapy (**Figure 1**). Quantitative perfusion parameters (PI, TTP, and MTT) were analyzed using CEUS quantification software (**Figure 2**).

In the CRT+E group, VM rates significantly decreased over time (before-RT: 0.35 ± 0.12; during-RT: 0.37 ± 0.14; after-RT: 0.24 ± 0.09; P = 0.003), whereas the CRT group exhibited stable VM rates throughout treatment (before-RT: 0.33 ± 0.10; during-RT: 0.36 ± 0.09; after-RT: 0.31 ± 0.08; P = 0.232). Intergroup comparisons revealed no significant differences in VM rates at before-RT (P = 0.65) or during-RT (P = 0.72). However, after-RT VM rates were significantly lower in the CRT+E group compared to the CRT group (0.24 ± 0.09 vs. 0.31 ± 0.08; P = 0.027) (**Tables 3**, **4**).

**Table 3:** Comparison of contrast-enhanced ultrasound parameters between two groups.

CEUS parameter	Time	CRT+E (n=60)	CRT (n=60)	*P-value*
VM rates	before radiotherapy	0.35 ± 0.12	0.33 ± 0.10	0.510
	during radiotherapy	0.37 ± 0.14	0.36 ± 0.09	0.779
	after radiotherapy	0.24 ± 0.09	0.31 ± 0.08	0.027
PI	before radiotherapy	103.66 ± 69.84	121.11 ± 73.43	0.476
	during radiotherapy	75.18 ± 46.64	94.02 ± 93.99	0.454
	after radiotherapy	65.67 ± 36.53	74.69 ± 61.21	0.598
TTP	before radiotherapy	9.89 ± 4.22	8.75 ± 3.45	0.389
	during radiotherapy	12.87 ± 5.65	11.03 ± 10.13	0.508
	after radiotherapy	28.36 ± 7.69	11.28 ± 7.69	0.000
MTT	before radiotherapy	35.27 ± 16.47	29.22 ± 29.72	0.458
	during radiotherapy	50.96 ± 23.73	46.95 ± 46.15	0.746
	after radiotherapy	69.36 ± 37.63	41.35 ± 33.60	0.027

Data are presented as the mean ± SD. P-values were calculated by independent-samples t-test.

PI, peak intensity; TTP, time to peak; MTT, mean transit time.

**Table 4:** Intra-group comparison of contrast-enhanced ultrasound parameters between two groups.

CEUS parameter	CRT+E (n=60)			*P-value*	CRT (n=60)			*P-value*
	Before radiotherapy	During radiotherapy	After radiotherapy		Before radiotherapy	During radiotherapy	After radiotherapy	
VM rates	0.35 ± 0.12	0.37 ± 0.14	0.24 ± 0.09	0.003	0.33 ± 0.10	0.36 ± 0.09	0.31 ± 0.08	0.232
PI	103.66 ± 69.84	75.18 ± 46.64	65.67 ± 36.53	0.091	121.11 ± 73.43	94.02 ± 93.99	74.69 ± 61.21	0.224
TTP	9.89 ± 4.22	12.87 ± 5.65	28.36 ± 7.69	0.000	8.75 ± 3.45	11.03 ± 10.13	11.28 ± 7.69	0.570
MTT	35.27 ± 16.47	50.96 ± 23.73	69.36 ± 37.63	0.002	29.22 ± 29.72	46.95 ± 46.15	41.35 ± 33.60	0.371

Data are presented as the mean ± SD. P-values were calculated by repeated measure analysis of variance.

PI, peak intensity; TTP, time to peak; MTT, mean transit time.

In the CRT+E group, PI values progressively declined from before-RT to after-RT with corresponding values of 103.66 ± 69.84, 75.18 ± 46.64, and 65.67 ± 36.53, respectively, while the CRT group showed a similar trend (before-RT: 121.11 ± 73.43; during-RT: 94.02 ± 93.99; and after-RT: 74.69 ± 61.21, respectively). No statistically significant differences were observed between the two groups at the three distinct time points (all P>0.05) (refer to **Table 3**). Additionally, there was no statistical significance observed in the comparisons within each group (all P>0.05) (**Table 4**).

The CRT+E group exhibited a marked increase in TTP across treatment phases (before-RT: 9.89 ± 4.22; during-RT: 12.87 ± 5.65; after-RT: 28.36 ± 7.69; P<0.001), contrasting with stable TTP values in the CRT group (before-RT: 8.75 ± 3.45; during-RT: 11.03 ± 10.13; after-RT: 11.28 ± 7.69; P=0.57). Additionally, after-RT TTP was significantly higher in CRT+E versus CRT (28.36 ± 7.69 vs. 11.28 ± 7.69; P<0.001), despite comparable baseline (P=0.25) and mid-RT (P=0.38) values (**Tables 3**, **4**).

In the CRT+E group, MTT progressively increased from before-RT (35.27 ± 16.47 s) to after-RT (69.36 ± 37.63; P = 0.002). Conversely, the CRT group showed no significant temporal changes (before-RT: 29.22 ± 29.72; during-RT: 46.95 ± 46.15; post-RT: 41.35 ± 33.60; P = 0.371). Furthermore, intergroup comparisons shown that post-RT MTT was significantly prolonged in the CRT+E group versus the CRT group (69.36 ± 37.63 vs. 41.35 ± 33.60; P = 0.027) (**Tables 3**, **4**).

**Figure 3:**
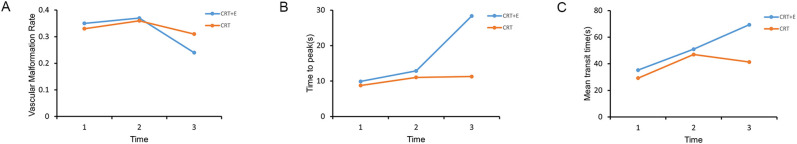
Line charts of DCE-US parameters in cervical cancer patients (n = 60 per group). **(A)** Changes in the vascular malformation (VM) rate before, during, and after radiotherapy in the CRT and CRT+E groups, illustrating the reduction in VM over time in response to treatment. **(B)** Time to peak (TTP) variations in both groups at different treatment stages, highlighting significant post-treatment differences between the CRT and CRT+E groups. **(C)** Mean transit time (MTT) trends in the CRT and CRT+E groups, showing a progressive increase in MTT in the CRT+E group, indicative of altered tumor perfusion dynamics.

In summary, after radiotherapy, both TTP and MTT values in the CRT+E group were significantly higher than those in the CRT group, and they displayed a gradually increasing trend (**Figure 3**).

## 4 Discussion

CCRT remains the standard treatment approach for LACC. A comprehensive meta-analysis evaluating the efficacy of platinum-based chemoradiotherapy has confirmed that the effectiveness has consistently stagnated, with a progression-free survival rate of only 58% and an approximate overall survival rate of 66% (2, 21). No survival benefit was noted with alterations in chemotherapy modes, such as the addition of adjuvant chemotherapy and induction chemotherapy, with the efficacy even lower than that of CCRT (22). The search for a more effective and low-toxicity treatment is imperative to enhance both efficacy and the quality of life for patients managing LACC. In recent years, angiogenesis inhibitors combined with standard radiotherapy and platinum-based chemotherapy have become a new focus in the treatment of advanced cervical cancer. Anti-angiogenic agents inhibit endothelial cell proliferation, hinder the formation of tumor angiogenesis, and cut off nutrient supply network for tumor growth, so as to achieve the aim of “starvation” tumor cells. More importantly, they can temporarily stabilize abnormal tumor blood vessels by regulating the balance of angiogenic factors, so as to normalize the irregular tumor vascular structures. This reduces vessel diameter and permeability, improves oxygen delivery and overall vascular reactivity (23). As an anti-angiogenic drug, Endostar can block angiogenesis and directly kill tumor cells. Besides, Endostar can also improve systemic chemotherapy by increasing tumor perfusion (24) and optimizing the hypoxic environment to increase radiotherapy sensitivity (8). Undoubtedly, undertaking a clinical evaluation of vascular normalization to optimize the synergistic effects achieved by combining Endostar with radiotherapy and chemotherapy is both crucial and challenging.

In terms of adverse reactions, this study demonstrates the absence of statistically significant differences in hematologic toxicity, hepatic and renal function, gastrointestinal reactions, and cardiac toxicity between the two groups (P>0.05). These adverse reactions improved following general symptomatic support treatments, including subcutaneous injection of recombinant granulocyte colony-stimulating factor, antiemetics, and antidiarrheals, while not interrupting the treatment course. Common adverse reactions in both groups consisted of bone marrow suppression and gastrointestinal reactions, mainly falling within Grade 1-2, with no instances of observed cardiac toxicity. Bevacizumab is a monoclonal antibody that specifically targets the vascular endothelial growth factor (VEGF) signaling pathway with high affinity for VEGF binding, effectively blocking this pathway. This inhibition disrupts VEGF binding to vascular endothelial growth factor receptor (VEGFR) on the surface of vascular endothelial cells, leading to the suppression of tumor angiogenesis, growth, and metastasis, making it an efficient anti-tumor agent. However, the use of bevacizumab is associated with substantial adverse reactions. A study involving 452 patients with advanced cervical cancer (25), indicated that combining bevacizumab with chemotherapy resulted in an increased occurrence of adverse events, including Grade 3 or higher thrombosis or embolism, Grade 2 or higher GI fistula, grade 2 or higher hypertension, Grade 4 or higher neutropenia, and genitourinary complications compared to chemotherapy alone. The most prevalent adverse reaction observed was grade 4 or higher neutropenia, with an incidence rate of 36%, followed by hypertension. Bevacizumab’s lack of selectivity for endothelial cells in both normal and tumor vasculature necessitates caution, particularly in hypertensive patients during treatment. Regular blood pressure monitoring is advisable, and antihypertensive drugs may be required. Additionally, the occurrence rate of thrombosis significantly increases due to endothelial dysfunction and exposure of subendothelial collagen caused by impaired endothelial function and defects. Therefore, patients with a history of thrombosis or embolism should also exercise caution. The relatively high occurrence of adverse reactions and the cost of bevacizumab limit its application in clinical practice for cervical cancer treatment. On the other hand, Endostar, belonging to the category of multi-targeted anti-angiogenic agents, effectively inhibits the migration of vascular endothelial cells. This study demonstrates that when used in combination with chemoradiotherapy, Endostar’s acute adverse reactions were overall mild, with no significant increase in the adverse effects of chemoradiotherapy. It proved to be well-tolerated and safe. Shu et al. conducted a randomized controlled trial that confirmed the ability of Endostar to significantly enhance the complete response (CR) rate when combined with CCRT in locally advanced cervical squamous cell carcinoma. The adverse reactions in this context were manageable and mild (9), consistent with the results of this study.

While microvessel density (MVD), vascularity, basement membrane, and pericyte coverage serve as gold standards for evaluating angiogenesis and vascular normalization (26), their clinical applications pose challenges due to their invasive nature and reliance on qualified biopsies. Previous studies have employed immunohistochemical (IHC) staining, CT perfusion imaging (CTPI), or dynamic contrast-enhanced MRI (DCE-MRI) to assess vascular normalization (27–29). Nonetheless, these approaches come with drawbacks such as ionizing radiation, time-consuming procedures, high costs, poor reproducibility, and inconvenience, potentially limiting their routine clinical utilization. In this study, transrectal contrast-enhanced ultrasound was used to observe the changes of tumor vessel density and blood flow before, during and after radiotherapy inpatients, which is simple, low-cost, radiation free, noninvasiveness, and beneficial to clinical application (14, 15). CEUS is an emerging technology in ultrasound diagnostics in recent years. The contrast technology and contrast agent used in CEUS can reflect the changes of blood perfusion characteristics of diseased tissues, and the microcirculatory perfusion status of diseased tissues can be reflected by the number of contrast agent microbubbles reaching diseased tissues and the speed of their entry and exit, namely the time-velocity relation curve (30). Moreover, as a non-invasive imaging method, CEUS has the potential to continuously monitor and evaluate the effect and progress of tumor vascular normalization induced by angiogenesis therapy. Referring to the introduction of CEUS as formulated by the European Federation of Societies for Ultrasound in Medicine and Biology (EFSUMB), hemodynamic indicators can be reflected by CEUS related parameters such as PI, TTP and MTT (31). The maximum value of the ultrasonic signal intensity in the time-velocity curve of the region of interest (ROI) is denoted as PI, which is closely associated with the number of intravascular microvesicles and reflects the blood volume in the microvessels of the ROI. The time taken for the signal intensity in the region of interest to go from the base value to the maximum value is referred to as TTP, while the average time required for the ultrasonic contrast agent to traverse the region of interest is termed MTT. Both TTP and MTT offer insights into the blood flow velocity within the microvessels of the ROI. Additionally, it is noteworthy that MVD serves as one of the gold standards for assessing angiogenesis and vascular normalization (26). Relevant studies indicate that ultrasound contrast parameters such as PI are correlated with the tumor’s MVD, and these correlations were found to be notably different. Consequently, a comprehensive analysis of the dynamic variations in CEUS parameters allows for an understanding of changes in tumor blood flow and provides insights into the progression of vascular normalization to a certain extent (32–35). Perfusion parameters such as TTP and MTT provide quantitative measures of tumor vascularity, allowing real-time monitoring of treatment response and facilitating personalized therapy adjustments (36, 37).

In the current study, CEUS was performed on the patients, and it was found that the vascular malformation rate after radiotherapy in the CRT+E group was significantly lower than that in the CRT group (P=0.027), indicating that Endostar can reduce the rate of vascular malformation in cervical cancer. This finding is consistent with recent studies that suggest the potential of anti-angiogenic agents like Endostar in reducing abnormal vascularization in tumors (8, 38). Such studies have highlighted the beneficial role of angiogenesis inhibitors in the normalization of tumor vasculature, thereby improving the efficacy of radiotherapy. Although PI in the CRT+E group exhibited a downward trend after radiotherapy, the difference was not statistically significant. This may be due to tumor heterogeneity, as variations in vascular density and perfusion within cervical cancer lesions can lead to differential responses to anti-angiogenic therapy. Additionally, the sensitivity limitations of CEUS may have contributed to this finding, as PI measurements are influenced by factors such as ultrasonic attenuation, microbubble distribution, and operator-dependent variability (39). The significant increases in TTP and MTT before, during, and after radiotherapy in the CRT+E group indicate a gradual slowing of contrast agent transit through the tumor, suggesting the normalization of tumor vasculature. This finding is consistent with previous studies (40, 41) that reported similar alterations in contrast dynamics following angiogenesis inhibitor treatment. Before treatment, cervical cancer is typically characterized by disorganized vasculature, with high blood volume and arteriovenous shunting leading to the rapid passage of contrast agents. As Endostar exerts its anti-angiogenic effects, it reduces tumor microvessel density, decreases blood perfusion, and normalizes vasculature, resulting in a significant reduction in blood flow velocity, as reflected by the observed increases in TTP and MTT. Our findings are consistent with prior research demonstrating that angiogenesis inhibitors can normalize abnormal vasculature in cervical cancer, thereby enhancing overall treatment efficacy (38, 42). These results contribute to the growing body of evidence supporting the role of anti-angiogenic agents in optimizing cancer therapies. However, despite previous studies establishing an association between CEUS parameters and MVD (43), direct histopathological validation was not performed in this study. While CEUS provides valuable insights into tumor perfusion, histopathological confirmation remains essential for validating microvascular density-related imaging findings (44). This limitation underscores the need for future research integrating immunohistochemical MVD quantification to corroborate CEUS results.

## 5 Limitations and prospect

Several limitations of this study should be acknowledged. First, the accuracy of DCE-US is influenced by both the operator’s expertise and the quality of the ultrasound equipment. Second, the relatively small sample size may have limited the statistical power of the study. Additionally, direct histopathological validation was not performed, and the follow-up period primarily focused on short-term treatment effects rather than long-term efficacy or survival outcomes. This focus was due to the study’s primary objective of evaluating the early anti-angiogenic effects of Endostar using contrast-enhanced ultrasound. However, we recognize the need for long-term follow-up as a limitation, and future studies are planned to assess disease progression and survival outcomes. Larger-scale studies with expanded cohorts may provide more robust evidence to validate the anti-angiogenic effects in LACC. Combining transrectal CEUS assessment with immunohistochemical MVD quantification could further strengthen the reliability of CEUS findings.

## 6 Conclusion

The combination of Endostar with CCRT for locally advanced cervical cancer (LACC) demonstrated favorable safety and tolerability, while long-term toxicity requires further follow-up. Transrectal contrast-enhanced ultrasound (CEUS) effectively assessed tumor vascular normalization induced by Endostar during CCRT. Specifically, Endostar significantly reduced VM rates and shortened MTT, suggesting its potential to normalize tumor vasculature. Long term follow up and a larger sample size are necessary for evaluating the clinical efficacy.

## Data Availability

The original contributions presented in the study are included in the article/supplementary material. Further inquiries can be directed to the corresponding author/s.
